# NADPH oxidase-4 promotes eccentric cardiac hypertrophy in response to volume overload

**DOI:** 10.1093/cvr/cvz331

**Published:** 2019-12-10

**Authors:** Moritz Schnelle, Iain Sawyer, Narayana Anilkumar, Belal A Mohamed, Daniel A Richards, Karl Toischer, Min Zhang, Norman Catibog, Greta Sawyer, Héloïse Mongue-Din, Katrin Schröder, Gerd Hasenfuss, Ajay M Shah

**Affiliations:** 1 King’s College London British Heart Foundation Centre of Excellence, School of Cardiovascular Medicine & Sciences, The James Black Centre, 125 Coldharbour Lane, London SE5 9NU, UK; 2 Department of Cardiology and Pneumology, University Medical Center Göttingen, Robert-Koch-Strasse 40, 37075 Göttingen, Germany; 3 Institute for Clinical Chemistry, University Medical Center Göttingen, Robert-Koch-Strasse 40, 37075 Göttingen, Germany; 4 DZHK (German Centre for Cardiovascular Research), Partner Site Göttingen, Göttingen, Germany; 5 Institute for Cardiovascular Physiology, Goethe-University, Theodor-Stern-Kai 7, 60596 Frankfurt am Main, Germany

**Keywords:** NADPH oxidase, Volume overload, Heart, Mouse models, Cardiac remodelling

## Abstract

**Aims:**

Chronic pressure or volume overload induce concentric vs. eccentric left ventricular (LV) remodelling, respectively. Previous studies suggest that distinct signalling pathways are involved in these responses. NADPH oxidase-4 (Nox4) is a reactive oxygen species-generating enzyme that can limit detrimental cardiac remodelling in response to pressure overload. This study aimed to assess its role in volume overload-induced remodelling.

**Methods and results:**

We compared the responses to creation of an aortocaval fistula (Shunt) to induce volume overload in *Nox4*-null mice (Nox4^−/−^) vs. wild-type (WT) littermates. Induction of Shunt resulted in a significant increase in cardiac Nox4 mRNA and protein levels in WT mice as compared to Sham controls. Nox4^−/−^ mice developed less eccentric LV remodelling than WT mice (echocardiographic relative wall thickness: 0.30 vs. 0.27, *P* < 0.05), with less LV hypertrophy at organ level (increase in LV weight/tibia length ratio of 25% vs. 43%, *P* < 0.01) and cellular level (cardiomyocyte cross-sectional area: 323 µm^2^ vs. 379 μm^2^, *P* < 0.01). LV ejection fraction, foetal gene expression, interstitial fibrosis, myocardial capillary density, and levels of myocyte apoptosis after Shunt were similar in the two genotypes. Myocardial phospho-Akt levels were increased after induction of Shunt in WT mice, whereas levels decreased in Nox4^−/−^ mice (+29% vs. −21%, *P* < 0.05), associated with a higher level of phosphorylation of the S6 ribosomal protein (S6) and the eIF4E-binding protein 1 (4E-BP1) in WT compared to Nox4^−/−^ mice. We identified that Akt activation in cardiac cells is augmented by Nox4 via a Src kinase-dependent inactivation of protein phosphatase 2A.

**Conclusion:**

Endogenous Nox4 is required for the full development of eccentric cardiac hypertrophy and remodelling during chronic volume overload. Nox4-dependent activation of Akt and its downstream targets S6 and 4E-BP1 may be involved in this effect.

## 1. Introduction

Cardiac remodelling in response to haemodynamic overload varies depending upon the nature of overload. Chronic pressure overload (e.g. with aortic stenosis or hypertension) induces concentric left ventricular (LV) remodelling, whereas chronic volume overload (e.g. with aortic or mitral regurgitation) induces eccentric LV remodelling.^1^ Previous studies show that distinct signalling pathways are involved in these responses. Chronic volume overload in the short to medium term may result in a more functionally compensated cardiac phenotype than chronic pressure overload at similar levels of overload or hypertrophy. Consistent with this notion, mice subjected to transverse aortic constriction (TAC) developed greater interstitial fibrosis, myocyte apoptosis, inflammation, systolic dysfunction, and mortality in the early stages after imposition of pressure overload than animals subjected to matched chronic volume overload.^2^ This was related to an increased activation of Akt in volume overload vs. calcium/calmodulin-dependent protein kinase II in pressure overload, indicating a differential activation of signalling pathways in the two settings. It has also been reported that alterations in Erk1/2 signalling differentially drive eccentric vs. concentric hypertrophy.^3^ The upstream drivers of such differential signalling in volume overload are not well understood.

Recent studies on the role of redox signalling pathways in cardiac remodelling have highlighted differential adaptive vs. detrimental effects depending upon the source of reactive oxygen species (ROS) and the context.^4^ NADPH oxidase (Nox) proteins have gained particular interest in this regard. In contrast to most other ROS sources, these enzymes generate ROS as their primary function and are implicated in specific redox signalling. Nox enzymes generate ROS by catalyzing electron transfer from NADPH to molecular oxygen. Of the seven known Nox isoforms, Nox2 and Nox4 are the predominant proteins expressed in the heart.^5^ Nox2 is activated by agonists such as angiotensin II (Ang II), whereas Nox4 is constitutively active and regulated mainly by its level of abundance. Nox2 is involved in augmenting maladaptive cardiac remodelling during chronic pressure overload or after myocardial infarction, through multiple mechanisms including an enhancement of Ang II-dependent cardiomyocyte hypertrophy, interstitial fibrosis, myocyte cell death, and abnormalities of calcium homeostasis.^6^ In contrast, Nox4 was found to have protective effects against chronic pressure overload-induced remodelling in mice, through a number of mechanisms including paracrine effects on myocardial capillary density^7^ and the activation of the cytoprotective transcription factor nuclear factor erythroid-derived 2-like 2 (Nrf2).^8^ The role of Nox4 in chronic volume overload has not previously been investigated. In this study, we investigated the role of Nox4 in the response to aortocaval fistula (Shunt), a model of chronic volume overload in mice.

## 2. Methods

### 2.1 Animals

Studies were conducted in accordance with the UK Home Office Guidance on the Operation of the Animals (Scientific Procedures) Act, 1986 and were approved by the institutional ethics committee. Global *Nox4*-null mice (Nox4^−/−^) were described previously and were on a C57Bl/6 background.^7^ Nox4^−/−^ mice were compared with wild-type (WT) littermates. Naïve WT mice were purchased from Harlan Laboratories (UK). Euthanization was performed under 2% isoflurane anaesthesia by intracardiac injection of 200 µL 5% potassium chloride (KCl) to induce cardiac arrest. All experiments were performed in male mice aged 7–12 weeks in order to avoid any confounding effects related to oestrogen cycle.

### 2.2 Animal surgery

Aortocaval fistula (Shunt) surgery was performed as described previously.^2^ In brief, mice were anaesthetised using 1.5% isoflurane insufflation. The aorta and inferior vena cava were dissected free from surrounding tissue through a longitudinal abdominal incision. The aorta was clamped just above the renal arteries and punctured with a 23 gauge needle through the inferior vena cava in an infrarenal position. After removing the needle, the external hole in the aorta was closed using cyanoacrylate glue. Mixing of oxygenated blood from the abdominal aorta into the vena cava could then be observed before the abdomen was closed. Finadyne s.c. and Vetergesic i.m. were administered for post-surgical analgesia. Sham animals underwent the same procedure except for puncture of vessels. Mice were kept on a heating plate until full recovery from anaesthesia. Studies were performed 2 weeks after surgery.

Minimally invasive TAC was performed under 1.5% isoflurane anaesthesia as described previously.^9^ A 6/0 suture was used to constrict the aortic arch around a 27 gauge needle. Sham animals underwent a similar procedure except for aortic constriction. Experiments were performed 2 weeks after surgery.

### 2.3 Echocardiography

Imaging was performed under 1.5% isoflurane anaesthesia on a heated platform using a Vevo 2100 Imaging System with a 40 MHz linear probe (Visualsonics, Canada).^10^ The relative wall thickness (RWT) in diastole was calculated as RWT = (septal wall thickness + posterior LV wall thickness)/LV diameter. Data analysis was performed with the Vevo^®^2100 software v.1.2.1 (Visualsonics).

### 2.4 Cell culture

H9C2 cells (rat cardiac-derived myoblasts) were purchased from ATCC (UK). Cells were cultured in DMEM supplemented with 100 U/mL penicillin, 100 µg/ml streptomycin, 2 mM glutamate, and 10% foetal calf serum (FCS) at 37°C/5% CO_2_. Primary cardiomyocytes were isolated from 1- to 2-day-old neonatal Sprague-Dawley rats as described previously.^11^ For manipulation of Nox4 levels, cells were allowed to adhere for 24 h before transduction with adenoviruses expressing Nox4 or β-galactosidase (β-Gal) at an MOI of 20.^7^ siRNA-mediated knockdown of Nox4 or PP2Ac was achieved using commercially available pre-validated sequences from Ambion (USA) with siPORT transfection reagent (Ambion) according to the manufacturer’s instructions. Cells were grown for 48 h in DMEM/10% FCS after adenoviral transduction or 72 h following siRNA transfection, respectively, before harvesting or further treatment. Cells were serum starved for 4 h before stimulation with insulin-like growth factor-I (IGF-I; 10 ng/mL). Src kinase inhibition was achieved by treatment with SU6656 (1 µM), a specific Src inhibitor,^12^ for 3 h. Treatment with DMSO served as control.

### 2.5 RT–PCR

Total RNA was isolated from LV tissue and cDNA was synthesized using Oligo(dTs) and M-MLV reverse transcriptase (Promega, UK). Quantitative RT**–**PCR was performed with the StepOnePlus™ System (Applied Biosystems, UK) using SYBR Green. Delta delta Ct values were calculated using GAPDH as a reference gene. Primer sequences are shown in Supplementary material online, *Table S1*.

### 2.6 Western blotting

Snap-frozen LV tissue samples were homogenized and lysed in a buffer containing: 25 mM Tris–HCl, 150 mM NaCl, 2 mM EGTA, 5 mM EDTA, 0.5% NP-40 with protease and phosphatase inhibitor cocktails (Sigma-Aldrich, UK). For cell lysates, the buffer composition was: 25 mM Tris–HCl, 150 mM NaCl, 0.5% Triton X-100, 0.1% SDS, and 0.1% sodium deoxycholate with protease and phosphatase inhibitor cocktails. Protein concentration was estimated using Bradford reagent (Sigma-Aldrich, UK). Tissue homogenates were separated by SDS/PAGE and transferred onto nitrocellulose membranes. Membrane fractions were obtained by centrifugation of heart lysates^13^ and further processed as described earlier. The following primary antibodies were used: phospho-Akt (S473), total Akt, phospho-Erk1/2 (Thr202/Tyr204), total Erk1/2, phospho-ribosomal protein S6 (S235/236), total ribosomal protein S6, phospho-Src (Tyr416), total Src, total PP2Ac (all Cell Signaling); Nox2 (BD Transduction); Nox4^14^; eIF4E-BP1, caveolin-3 and phospho-PP2Ac (Tyr307) (Abcam); p47phox (EMD Millipore/Upstate); GAPDH and β-actin (Sigma). Imaging and densitometric quantification were undertaken either using enhanced chemiluminescence or with an Odyssey Li-Cor imaging system (Li-Cor Biosciences, UK).

### 2.7 Histology

Hearts were arrested in diastole and perfusion fixed in 4% paraformaldehyde. About 6 µm transverse sections were used to quantify LV cardiomyocyte cross-sectional area after staining with FITC-conjugated wheat germ agglutinin (FITC-WGA, Vector RL-1022). At least 50 cardiomyocytes per section were analysed. Capillary density (capillaries per mm^2^) was measured after isolectin B4 immunostaining (Vector B-1205),^7^ and apoptosis by TUNEL-staining (Millipore S7110 kit). Interstitial fibrosis was quantified in Picrosirius red-stained sections. Image analysis was performed using Fiji image analysis software or Volocity (Perkin Elmer, UK).

### 2.8 Glutathione assay

Glutathione measurements were performed in snap-frozen LV tissue using the GSH-Glo assay kit (Promega, UK) according to the manufacturer’s protocol. Reduced glutathione (GSH) was measured in the lysate while total glutathione (GSH+GSSG) was quantified after pre-incubation of the lysate in 1 mM Tris (2-carboxyethyl) phosphine hydrochloride (TCEP; Sigma-Aldrich, UK). Oxidized glutathione levels (GSSG) and the ratio of reduced to oxidized glutathione (GSH/GSSG) were calculated afterwards.

### 2.9 Statistical analysis

Data are presented as mean ± SEM. Unpaired Student’s *t*-tests were undertaken for pure WT comparisons. For all other experiments, one-way or two-way ANOVA followed by Bonferroni test for multiple comparisons was used. *P* < 0.05 was considered statistically significant. Analyses were performed using GraphPad Prism v6.

## 3. Results

### 3.1 Volume overload increases myocardial Nox4 but not Nox2 levels

**Figure 1:**
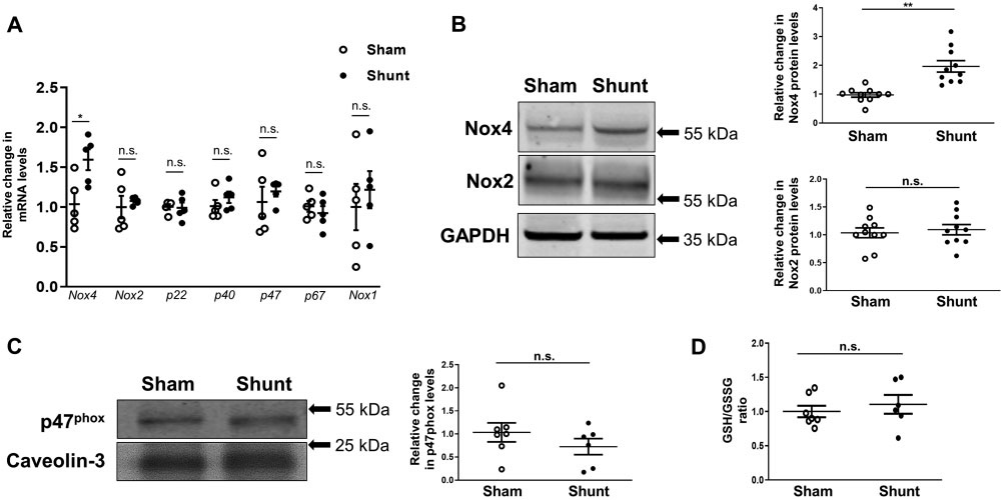
LV expression of NADPH oxidases and glutathione redox status in WT hearts after 2 weeks of volume overload. (*A*) LV mRNA levels of Nox4, Nox2, Nox subunits (p22^phox^, p40^phox^, p47^phox^, and p67^phox^), and Nox1 after Shunt compared to respective Sham controls. GAPDH mRNA was used for normalization; *n* = 5/group. (*B*) LV protein levels of Nox4 and Nox2 after Shunt compared to Shams; *n* = 10/group. (*C*) Protein levels of p47^phox^ in the LV membrane-enriched fraction, as a marker of Nox2 activation. Caveolin-3 was used as a membrane marker and loading control (*n* = 6-7/group). (*D*) Reduced (GSH) over oxidized (GSSG) glutathione ratio in LV lysates after Shunt compared to Sham; (*n* = 6–7/group). *P < 0.05, **P < 0.01, n.s., not significant between Sham and Shunt by unpaired Student’s *t*-test.

Two weeks of volume overload in WT mice caused a significant increase in Nox4 mRNA levels (1.6-fold, *P* < 0.05) and protein levels (2.0-fold, *P* < 0.01) in left ventricle as compared to Sham controls (*Figure 1A *and* B*). No changes were found in the levels of Nox2, the oxidase subunits p22^phox^, p40^phox^, p47^phox^ and p67^phox^, or Nox1 (*Figure 1A *and* B*). The specificity of the Nox4 and Nox2 antibodies is shown in Supplementary material online, *Figure S1*. Using membrane enrichment of p47^phox^ as an index of Nox2 activation, there was no significant change after induction of Shunt (*Figure 1C*). We assessed the reduced-to-oxidized glutathione ratio in the left ventricle and found no change after imposition of volume overload (*Figure 1D*), indicating a well-preserved overall redox state.

In contrast to Shunt, TAC resulted in a significant increase in LV mRNA levels of Nox2 (2.4-fold, *P* < 0.05), p40^phox^ (2.1-fold, *P* < 0.01), and p47^phox^ (2.1-fold, *P* < 0.01) as well as Nox4 (3.2-fold, *P* < 0.05)—Supplementary material online, *Figure* S*2A*. In addition, there was a significant decrease in reduced-to-oxidized glutathione ratio after TAC (0.73 vs. 1.0 in Sham hearts, *P* < 0.05)—Supplementary material online, *Figure S2B*.

### 3.2 Nox4 is required for full eccentric LV remodelling after volume overload

**Figure 2:**
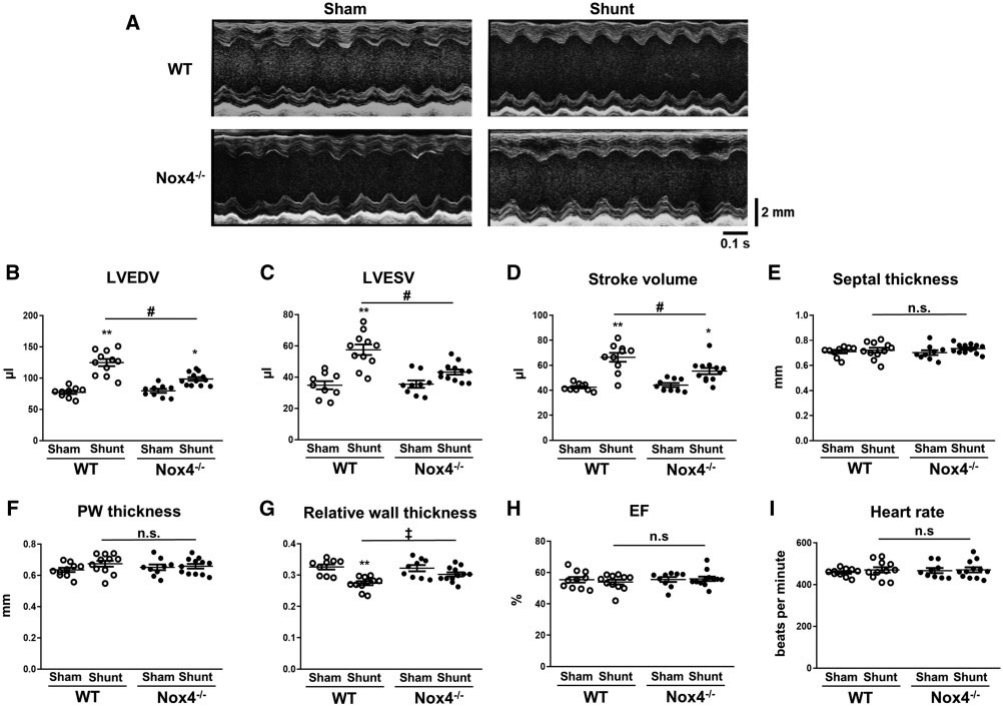
Cardiac dimensions and function in Nox4^−/−^ mice and WT littermates after volume overload. (*A*) Representative LV echocardiographic M-mode images after Shunt compared to Sham control; scale bar in mm on the right, time stamp in seconds at the bottom. (*B*–*I*) Mean data of LV end-diastolic volume (LVEDV) (*B*), end-systolic volume (LVESV) (*C*), stroke volume (*D*), septal thickness (*E*), posterior wall (PW) thickness (*F*), relative wall thickness (*G*), ejection fraction (EF) (*H*), and heart rate (*I*) after Shunt compared to Sham (*n* = 9–12/group). *P < 0.05, **P < 0.01 for Shunt vs. respective Sham controls, ^‡^*P* < 0.05 significant interaction between genotypes, ^#^*P* < 0.01 between Shunt groups and significant interaction (*P* < 0.01) between genotypes, n.s., not significant between genotypes using two-way ANOVA followed by Bonferroni *post-hoc* test for multiple comparisons.

Cardiac remodelling following Shunt or Sham was assessed by echocardiography. Representative echocardiographic M-mode images are shown in *Figure 2A*. Quantitative analysis revealed that Nox4^−/−^ mice developed less LV dilatation after Shunt than WT littermates, with smaller LV diastolic volumes (+23.8% vs. +61.4%, *P* < 0.01) and LV systolic volumes (+21.7% vs. +65.1%, *P* < 0.01)—*Figure 2B *and* C*. As such, LV stroke volume was significantly higher in WT mice than Nox4^−/−^ after Shunt (*Figure 2D*). The septal and posterior wall thickness were similar in Nox4^−/−^ and WT mice (*Figure 2E *and* F*). As a consequence, relative wall thickness decreased to a greater extent in WT mice than Nox4^−/−^ animals (*Figure 2G*), indicative of more eccentric remodelling. There was no change in ejection fraction after Shunt in either WT or Nox4^−/−^ mice, while heart rates were also similar among groups (*Figure 2H *and* I*). The lung weight/tibia length ratio was unaltered after Shunt both in WT mice (9.02 vs. 8.68 mg/mm in Sham, *P* = n.s.) and Nox4^−/−^ mice (9.12 vs. 8.43 mg/mm in Sham, *P* = n.s.), indicating an absence of pulmonary congestion.

### 3.3 Nox4 contributes to cardiomyocyte hypertrophy during chronic volume overload but does not affect fibrosis or capillary density

**Figure 3:**
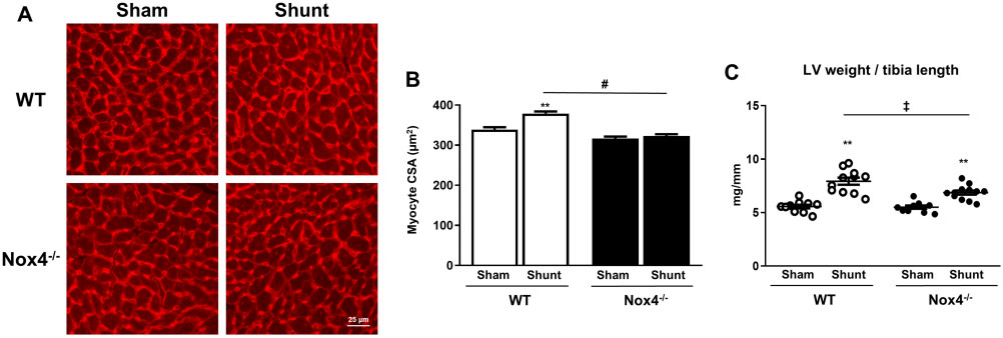
Cardiac hypertrophy in Nox4^−/−^ mice and WT littermates after volume overload. (*A*) Representative histological images showing LV myocardial sections stained with wheat germ agglutinin (WGA) after Shunt or Sham. (*B*) Myocyte cross-sectional area (CSA)—mean ± SEM for ≥350 cells and 6–7 hearts/group. (*C*) LV weight vs. tibia length (TL) ratio after Shunt or Sham (*n* = 10–12/group). **P < 0.01 for Shunt vs. Sham, ^‡^*P* < 0.05 between Shunt groups and significant interaction (*P* < 0.05) between genotypes, ^#^*P*< 0.01 between Shunt groups and significant interaction (*P* < 0.01) between genotypes using two-way ANOVA followed by Bonferroni *post-hoc* test for multiple comparisons.

The cardiomyocyte cross-sectional area in LV sections after 2 weeks of volume overload was significantly smaller in Nox4^−/−^ compared to WT littermate mice (322.8 µm^2^ vs. 378.8 μm^2^, *P* < 0.01) (*Figure 3A *and* B*). This was also evident at a macroscopic level as the LV weight/tibia length ratio increased to a lesser extent in Nox4^−/−^ mice after Shunt than in WT littermates (+ 25% vs. +43%, *P* < 0.05)—*Figure 3C*. Body weights, right atrial, right ventricular, and left atrial weights were similar in Nox4^−/−^-mice and WT littermates after volume overload (Supplementary material online, *Figure S3A–D*).

**Figure 4:**
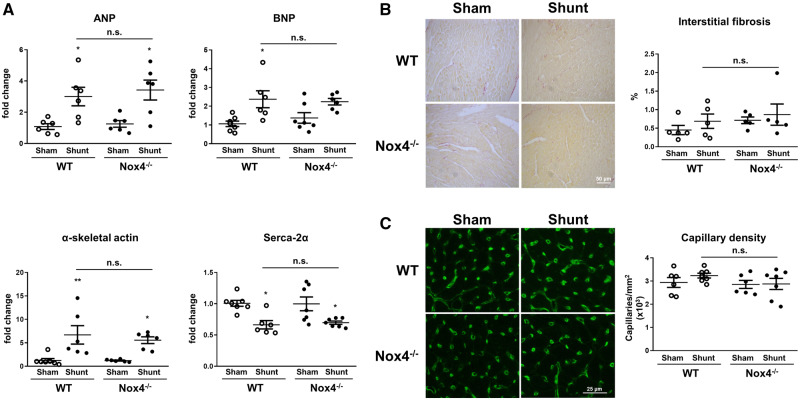
Cardiac gene expression, fibrosis, and capillary density in Nox4^−/−^ mice and WT littermates after volume overload. (*A*) LV mRNA levels of ANP (atrial natriuretic peptide), BNP (brain natriuretic peptide), α-skeletal actin, and SERCA-2α (sarcoplasmic/endoplasmic reticulum calcium ATPase-2α) after Shunt or Sham control. (*B* and *C*) Representative images and mean data of Picrosirius red-stained sections indicating fibrotic regions (*B*) and isolectin B4-staining depicting cardiac capillaries (*C*) after Shunt or Sham (*n* = 5–7/group). *P < 0.05, **P < 0.01 in Shunt vs. Sham, n.s., not significant between genotypes using two-way ANOVA followed by Bonferroni *post-hoc* test for multiple comparisons.

Nox4^−/−^ mice and WT littermates showed similar changes in LV mRNA levels of ANP (3.4-fold vs. 3.0-fold, *P* = n.s.), BNP (2.2-fold vs. 2.4-fold, *P* = n.s.), α-skeletal actin (5.6-fold vs. 6.7-fold, *P* = n.s.), and SERCA-2α (0.7-fold vs. 0.7-fold, *P* = n.s.) (*Figure 4A*). There was no increase in interstitial fibrosis or any change in capillary density after Shunt in either genotype (*Figure 4B *and* C*). There was also no significant increase in apoptosis as assessed by TUNEL staining in either group (Supplementary material online, *Figure* S*4A* and *B*).

### 3.4 Nox4 enhances Akt signalling during volume overload

**Figure 5:**
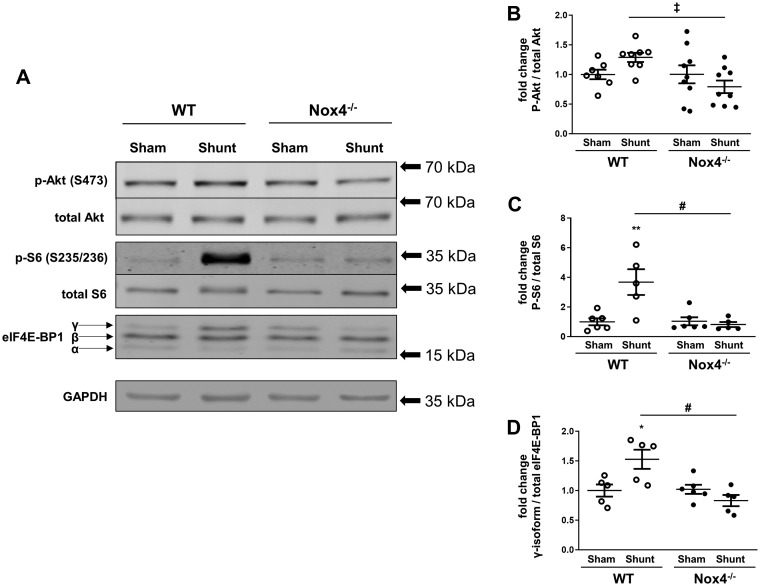
Phosphorylation of Akt and downstream targets in Nox4^−/−^ mice and WT littermates after volume overload. (*A*) Representative western blot images for phospho-Akt at Ser^473^ (p-Akt), total Akt, phospho-S6 ribosomal protein (p-S6), total S6 ribosomal protein, eIF4E-BP1 (eukaryotic translation initiation factor 4E-binding protein 1; divided into α, β, and γ-isoforms), and GAPDH as loading control in the LV after Shunt and Sham. (*B*–*D*) Mean data for phosphorylated vs. total Akt (*B*), phosphorylated vs. total S6 ribosomal protein (*C*), and the hyper-phosphorylated γ-isoform vs. total eIF4E-BP1 (*D*) using densitometry (*n* = 5–9/group). *P < 0.05, **P < 0.01 in Shunt vs. Sham, ^‡^*P* < 0.05 between Shunt groups and significant interaction (*P* < 0.05) between genotypes, ^#^*P* < 0.01 between Shunt groups and significant interaction (*P* < 0.01) between genotypes using two-way ANOVA followed by Bonferroni *post-hoc* test for multiple comparisons.

Since Akt activation was shown to be important in the LV remodelling response to volume overload,^2^^,^^15^ we quantified phospho-Akt levels (Ser^473^) in LV tissue of Nox4^−/−^ mice and WT littermates after Shunt as compared to their respective Sham controls. We found that phospho-Akt levels were significantly lower after Shunt in Nox4^−/−^ mice than WT (*Figure 5A *and* B*). The downstream targets of Akt/mTOR signalling that are involved in protein synthesis, namely the S6 ribosomal protein and eIF4E-BP1 (eukaryotic translation initiation factor 4E-binding protein 1)^16^ were also analysed. In line with the results of Akt activation, S6 ribosomal protein phosphorylation at Ser^235/236^ was significantly higher in the left ventricle of WT mice compared to Nox4^−/−^ animals after Shunt (*Figure 5A *and* C*) as was the hyper-phosphorylated γ-isoform of eIF4E-BP1 (*Figure 5A *and* D*).

Changes in the activation of Erk1/2 were also previously implicated in eccentric remodelling.^3^ We found that the phosphorylation of Erk1/2 at Thr^202^/Tyr^204^ decreased in LV tissue after imposition of volume overload in WT as well as Nox4^−/−^-mice, but there was no difference between genotypes (Supplementary material online, *Figure* S*5A* and *B*).

### 3.5 Nox4 enhances Akt phosphorylation via Src-dependent inactivation of PP2A

**Figure 6:**
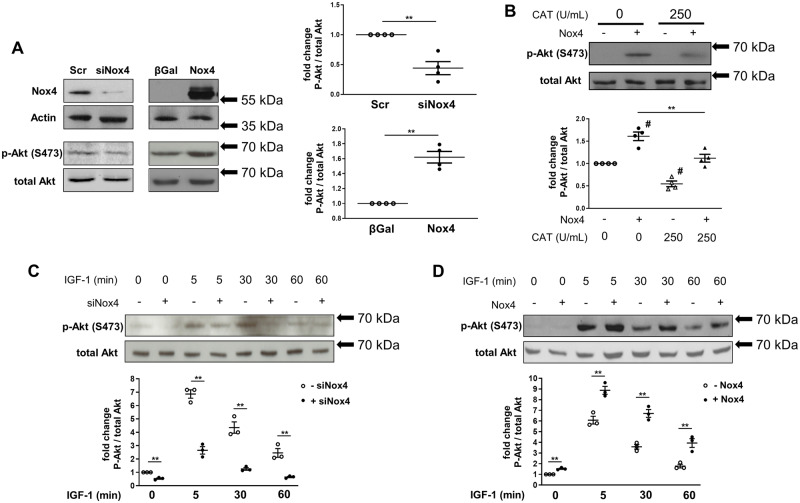
Nox4-dependent changes in Akt phosphorylation in cultured cardiac cells. (*A*) Protein levels of Nox4, actin as loading control, phospho (Ser^473^)-Akt (p-Akt), and total Akt were assessed by western blotting in lysates from H9C2 cells after siRNA-mediated knockdown of Nox4 (siNox4) or adenoviral-mediated Nox4 overexpression (Nox4). Scrambled siRNA (Scr) and overexpression of β-Gal were used as respective controls. Cells were cultured in 10% FCS containing medium. (*B*) Effects of PEG-catalase treatment (CAT 250 U/mL) or vehicle control (CAT 0 U/mL) on p-Akt levels following Nox4 (+Nox4) or control β-Gal overexpression (-Nox4). (*C* and *D*) Assessment of p-Akt and total Akt protein levels after stimulation with IGF-I (10 ng/mL) in Nox4 knockdown cells (+siNox4) or controls (-siNox4: scrambled siRNA was used) (*C*), and in cells with Nox4 overexpression (+Nox4) vs. control (-Nox4: transduction with a β-Gal expressing adenovirus) (*D*). Immunoblots were quantified by densitometry; data are the mean of 3–4 independent experiments. **P < 0.01 using unpaired Student’s *t*-test (*A*, *C*, *D*) and one-way ANOVA followed by Bonferroni *post-hoc* test for multiple comparisons (*B*) with # indicating *P* < 0.01 vs. control condition (-Nox4, 0 U/mL PEG-catalase).

To further investigate the mechanism underlying Nox4-dependent up-regulation of Akt activity, we turned to experiments in cultured cardiac cells. H9C2 cardiomyoblasts were stimulated with 10% FCS or IGF-I, which is implicated in responses to volume overload^17^ and stretch^18^ and induces Akt activation. In FCS-stimulated cells, the siRNA-mediated knockdown of endogenous Nox4 significantly reduced phospho-Akt levels (Ser^473^) as compared to a scrambled siRNA control (*Figure 6A*). On the other hand, adenoviral-mediated overexpression of Nox4 increased Akt activation assessed by Ser^473^ phosphorylation as compared to transduction with β-Gal (*Figure 6A*). The effects of Nox4 were inhibited in the presence of catalase (*Figure 6B*), indicating that they were ROS mediated. IGF-1 induced a time-dependent increase in Akt phosphorylation which was significantly attenuated when Nox4 was knocked down and significantly enhanced when Nox4 levels were increased (*Figure 6C *and* D*). These findings indicate that Nox4 augments Akt activation in cardiac cells similar to the findings in hearts after volume overload.

Redox-dependent enhancement of kinase activation is typically mediated by the inactivation of phosphatases that normally counteract activation, with the serine-threonine phosphatase PP2A (protein phosphatase 2A) being a likely candidate in this setting.^19^ To test the possible involvement of PP2A, we first investigated the effect of a phosphatase inhibitor, okadaic acid, at a dose (10 nM) that inhibits PP2A relatively selectively.^20^ Okadaic acid significantly increased Akt phosphorylation in FCS-stimulated H9C2 cells transduced with β-Gal but there was no additional effect in Nox4-overexpressing cells (Supplementary material online, *Figure* S*6A* and *B*). We confirmed that Nox4 was also involved in enhancing Akt activation in primary cardiomyocytes (Supplementary material online, *Figure S7A* and *B*) and in this system more directly assessed the involvement of PP2A. We found that the reduction in Akt phosphorylation observed when Nox4 was knocked down was reversed by the co-transfection of an siRNA that knocked down the PP2A catalytic subunit (Supplementary material online, *Figure* S*7A–C*). These data suggest that Nox4-mediated inhibition of PP2A is involved in the enhanced activation of Akt.

**Figure 7:**
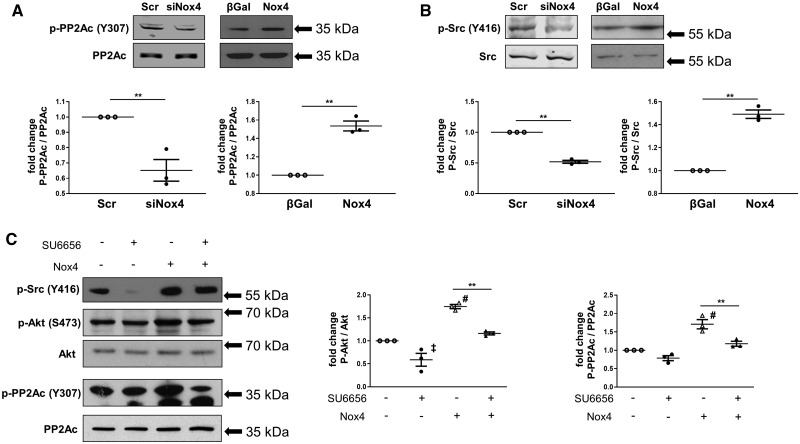
Role of Src kinase and PP2A phosphorylation in Nox4-dependent Akt activation in cardiac cells. (*A* and *B*) Protein levels of phospho (Tyr^307^)-protein phosphatase 2A catalytic subunit (p-PP2Ac), total PP2Ac (A), phospho (Tyr^416^)-Src (p-Src), and total Src (B) assessed by western blotting in lysates from H9C2 cells. Nox4 knockdown or overexpression was performed as in *Figure 6* (*n* = 3 independent experiments); **P < 0.01 using unpaired Student’s *t*-test. (*C*) Cells were transduced with Nox4 (+Nox4) or β-Gal (-Nox4) containing adenoviruses for 48 h followed by incubation with the Src inhibitor SU6656 (+SU6656; 1 µM) or DMSO (-SU6656) for 3 h. Protein levels were quantified by densitometry (*n* = 3 independent experiments). ^‡^*P* < 0.05, ^#^*P* < 0.01 vs. respective control (-Nox4, -SU6656), **P < 0.01 as indicated using one-way ANOVA followed by Bonferroni *post-hoc* test for multiple comparisons.

Potential mechanisms of PP2A inhibition include a variety of post-translational modifications of the enzyme, notably the phosphorylation of PP2Ac (the catalytic subunit) at Tyr^307^.^21^^,^^22^ We found that the knockdown of Nox4 was accompanied by a significant reduction in PP2Ac phosphorylation at Tyr^307^, whereas Nox4 overexpression significantly increased PP2Ac phosphorylation (*Figure 7A*). Total protein levels of PP2Ac were unaltered by manipulation of Nox4. PP2Ac phosphorylation at Tyr^307^ and its consequent inactivation may be driven by Src kinase, which itself is strongly redox activated^20^^,^^23^—making this a potential target of Nox4. Assessment of Src phosphorylation at Tyr^416^ (which reflects activation of the kinase) revealed that this was reduced when Nox4 was knocked down but was significantly increased upon Nox4 overexpression (*Figure 7B*). To investigate whether the Nox4-dependent changes in PP2Ac phosphorylation and Akt activation are the result of Src activation, we tested the effects of a specific Src inhibitor SU6656.^12^ This experiment showed that the significant increases in PP2Ac and Akt phosphorylation induced by overexpression of Nox4 were inhibited in the presence of SU6656 (*Figure 7C*). Taken together, these results suggest that Nox4-dependent enhancement of Akt activation involves the activation of Src which leads to phosphorylation and inactivation of PP2Ac, thereby relieving the brake on Akt phosphorylation (Supplementary material online, *Figure* S*8*).

## 4. Discussion

Nox4 expression levels are increased by diverse stresses, including hypoxia, starvation, myocardial ischaemia, and chronic pressure overload.^7^ Here, we demonstrate that *in vivo* chronic volume overload is also a potent stimulus for the induction of Nox4 in the heart. An increase in Nox4 levels is required for the development of eccentric LV chamber remodelling during volume overload since *Nox4*-null mice failed to develop the same extent of remodelling as did WT littermates. We also found a Nox4-dependent increase in Akt activation and downstream markers of protein synthesis (S6 ribosomal protein and eIF4E-BP1), as well as increased cardiomyocyte hypertrophy, suggesting that these changes may underlie the effect on eccentric remodelling. Indeed, previous findings showed that Akt activation was required for the development of eccentric LV remodelling after volume overload. Akt-deficient mice developed less cardiomyocyte hypertrophy and less eccentric remodelling during volume overload than WT mice and this was accompanied by worse heart failure in the longer term. Analogous to the Akt-dependent phenotype, the Nox4-dependent increase in eccentric LV remodelling after volume overload appears to be adaptive since WT mice had a higher stroke volume than *Nox4*-null mice. Taken together, these results suggest that an up-regulation of Nox4 contributes significantly to adaptive eccentric remodelling in response to volume overload, at least in part through an enhancement of myocardial Akt activation. These findings contrast to the effects of Nox4 during chronic pressure overload where it was found that Nox4 reduced the extent of concentric LV hypertrophy.^7^

Previous work in which murine pressure overload and volume overload matched for the extent of hypertrophy and wall stress were compared showed that volume overload was associated with compensated ventricular function in the short term, in contrast to pressure overload. We confirmed this finding in the current study and also analysed changes in myocardial redox state in the two settings. We found that pressure overload induced by TAC resulted in a more oxidized state as assessed by the GSH/GSSG ratio whereas redox state was well preserved during volume overload. In line with this finding, TAC was accompanied by a significant increase in expression levels of Nox2 oxidase subunits whereas this was not evident during volume overload. This finding reinforces previous data that Nox2 and Nox4 have contrasting regulation and mediate distinct signalling responses in the heart. Interestingly, it has been found previously that cardiomyocyte stretch results in the activation of Nox2,^24^ whereas we found no evidence of increased steady-state myocardial Nox2 activation during volume overload, which should induce cardiomyocyte stretch. However, we did not assess Nox2 activation at the level of the cardiomyocyte nor the responses to dynamic stretch in the current study.

Increased myocardial Akt activation is known to mediate adaptive functional responses in diverse settings. In addition to its role in mediating adaptation to volume overload as discussed earlier, Akt activation is involved in postnatal heart growth^25^ and in the development of physiological cardiac hypertrophy, with Akt^−/−^ mice failing to develop hypertrophy in response to swimming exercise.^26^ Interestingly, the chamber phenotype in physiological hypertrophy involves eccentric LV remodelling just as in volume overload. It was also shown that a cardiomyocyte-specific increase in Akt activation in mice leads to cardiac hypertrophy with enhanced function.^27^^,^^28^ Furthermore, Akt-deficient mice show an increase in maladaptive cardiac remodelling and contractile dysfunction in response to chronic pressure overload.^26^^,^^29^ In the current study, we show that Nox4 is an upstream activator of Akt during chronic volume overload since *Nox4*-null mice displayed less Akt activation than WT littermates following 2 weeks of Shunt and displayed a phenotype of reduced eccentric remodelling similar to that previously found in Akt-deficient mice.^15^ Akt may stimulate protein synthesis and cell growth through the activation of mTORC1 (mammalian target of rapamycin complex 1) which in turn phosphorylates several targets that are involved in regulating protein translation. Among these, the phosphorylation of S6 ribosomal kinase 1 and eIF4E-BP1 are implicated in enhancing the hypertrophic growth of cardiomyocytes.^25^ In relation to volume overload, the treatment of mice with an mTOR inhibitor led to a reduction in phosphorylation of the S6 ribosomal protein (a major target of the S6 ribosomal kinase 1) and eIF4E-BP1 and was accompanied by less eccentric remodelling and a higher mortality as compared to vehicle-treated animals.^17^ In line with a Nox4 augmentation of myocardial Akt activation in the current study, we also observed a strong Nox4 dependence of the S6 ribosomal protein and eIF4E-BP1 phosphorylation during volume overload.

Nox4 has previously been reported to enhance Akt activation in other cell types, such as adipocytes,^30^ pancreatic cancer cells,^31^ HEK293 cells,^14^ and vascular smooth muscle cells.^5^ These reports and the current results indicate a close relationship between Nox4 and Akt signalling; however, the underlying mechanisms have not hitherto been well defined. Our studies in cultured cardiac cells provide novel insights into the mechanistic underpinning of the link between Nox4 and Akt signalling. In line with the *in vivo* data in experimental volume overload, Akt phosphorylation was enhanced by Nox4 both in H9C2 cells and primary cardiomyocytes. We found that the enhancement of Akt phosphorylation was likely related to an inhibition of PP2A, which in turn was driven by a Nox4-dependent activation of Src and subsequent phosphorylation and inactivation of PP2A. Src is well recognized to be redox activated,^23^^,^^32^ and a previous report found that Nox4 can activate Src.^33^ Here, we show that endogenous Nox4 mediates Akt activation via a Src-PP2A axis. These findings add to the increasing recognition that the effects of Nox4 are typically driven through specific redox signalling rather than a generalized oxidative stress.^4^ In the setting of chronic volume overload, this mechanism (Nox4-dependent Akt activation) appears to be a key driver of the Nox4-dependent phenotype identified in the current study. With respect to Akt signalling, previous studies also reported that chronic persistent myocardial Akt activation may lead to pathological cardiac remodelling and systolic dysfunction.^34^ This possibility was not explored in the current study since we were using a Nox4-deficient mouse model; however, previous work in Akt-deficient mice subjected to chronic volume overload found a detrimental phenotype with increased mortality,^15^ suggesting that beneficial effects of Akt are maintained in the longer term during chronic volume overload.

Nox4 has been shown to have protective effects in many pathophysiological settings including during chronic pressure overload-induced remodelling,^7^^,^^8^ myocardial ischaemia–reperfusion,^13^^,^^35^ hind-limb ischaemia,^36^^,^^37^ kidney injury,^38^ remodelling after myocardial infarction,^39^ and atherosclerosis.^40–42^ Different Nox4-regulated mechanisms are reported to be involved in different settings. For example, a preservation of myocardial capillary density was found to be involved in the protective effects of Nox4 during chronic pressure overload—a setting in which pathological concentric remodelling is inhibited.^7^ However, in the current study, we found no differences in myocardial capillary density between *Nox4*-null and WT mice subjected to Shunt. In fact, Nox4 enhanced eccentric remodelling during Shunt. Another mechanism that has been related to the eccentric growth of cardiomyocytes *in vitro* is a reduction in Erk1/2-phosphorylation.^3^ The activation of Erk1/2 is known to be redox sensitive and was shown to be capable of being enhanced by Nox4 in HEK cells.^14^ However, we found no impact of Nox4 deletion on the levels of myocardial Erk1/2-phosphorylation after volume overload. These findings further support the idea that the effects of Nox4 involve specific redox signalling rather than global changes in redox state and suggest that Nox4-mediated Akt activation may be a major mechanism underlying the effects of Nox4 on volume overload-induced cardiac remodelling.

In summary, the present study shows that Nox4 plays an important role in the development of eccentric LV remodelling after the imposition of volume overload in mice. This may be mediated by a Nox4-dependent enhancement of Akt activation and signalling. The results add to the growing literature on diverse adaptive effects of Nox4 elevation and suggest that caution should be taken in the development of non-isoform-selective Nox inhibitors for different disease indications.

## Supplementary material

Supplementary material is available at *Cardiovascular Research* online.

## Authors’ contributions

A.M.S. and G.H. conceived the project. M.S. and A.M.S. designed experiments. M.S., I.S., N.A., B.A.M., D.A.R., M.Z., N.C., G.S., and H.M.D. performed experiments. K.S. provided essential reagents and advice. M.S., I.S., N.A., B.A.M., D.A.R., K.T., M.Z., N.C., H.M.D., G.H., and A.M.S. analysed and interpreted the data. M.S. and A.M.S. wrote the manuscript with further input from all authors.

**Conflict of** **interest:** none declared.

## Funding

This work was supported by the British Heart Foundation and the DFG (Deutsche Forschungsgemeinschaft) through the International Research Training Group Award IRTG1816 (to M.S.) and the Collaborative Research Center SFB1002 (to K.T. and G.H.).

## Supplementary Material

cvz331_Supplementary_Material

## Notes

**Time for primary review: 21 days**

## Notes

Translational perspective Cardiac volume overload resulting, for example, from aortic or mitral regurgitation contributes to the development of heart failure. Its underlying pathophysiology differs from that of cardiac pressure overload. Our study identifies the reactive oxygen species generating enzyme NADPH oxidase-4 (Nox4) as an important regulator of volume overload-induced cardiac remodelling by promoting eccentric left ventricular hypertrophy, an adaptive response to the increased volume. As Nox inhibition is currently being developed as a potential therapeutic approach for several human diseases (e.g. lung fibrosis), our findings highlight the importance of assessing the potential impact on cardiac function in patients with co-existent valvular regurgitation.
