# Supplementary material for: NADPH oxidase-4 promotes eccentric cardiac hypertrophy in response to volume overload
Source: Cardiovasc Res. 2019 Dec 10;117(1):178–87. doi: 10.1093/cvr/cvz331 (PMC7797217; doi:10.1093/cvr/cvz331)
Supplement: cvz331_Supplementary_Material [file cvz331_supplementary_material.pdf]

Supplementary Material

NADPH oxidase-4 promotes eccentric cardiac hypertrophy in response to volume overload

Moritz Schnelle[1,2,3,4], Iain Sawyer[1], Narayana Anilkumar[1], Belal A. Mohamed[2,4], Daniel A. Richards[1], Karl Toischer[2,4], Min Zhang[1], Norman Catibog[1], Greta Sawyer[1], Héloïse Mongue-Din[1], Katrin Schröder[5], Gerd Hasenfuss[2.4] & Ajay M. Shah[1]

[1] King's College London British Heart Foundation Centre of Excellence, School of Cardiovascular Medicine & Sciences, London, United Kingdom;

[2] Department of Cardiology and Pneumology, University Medical Center Goettingen, Germany;

[3] Institute for Clinical Chemistry, University Medical Center Goettingen, Germany;

[4] DZHK (German Centre for Cardiovascular Research), Partner Site Goettingen, Germany;

[5] Institute for Cardiovascular Physiology, Goethe-University, Frankfurt, Germany.

Correspondence to:

Professor Ajay M Shah

James Black Centre

King's College London

125 Coldharbour Lane

London SE5 9NU

UK

Tel: 44-2078485189 Fax: 44-2078485193

email: ajay.shah@kcl.ac.uk

Results

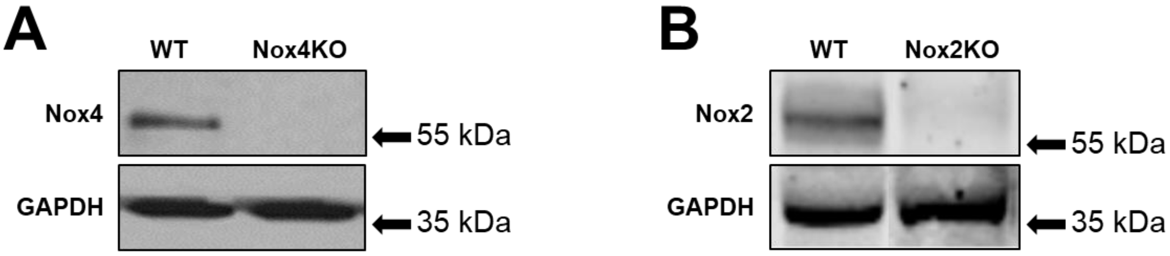

Suppl. Figure 1:

Specificity of Nox4 and Nox2 antibodies. Immunoblotting for Nox4 (A) and Nox2 (B) protein in heart lysates from WT and global Nox4 and Nox2 knockout mice (Nox4KO, Nox2KO) respectively.

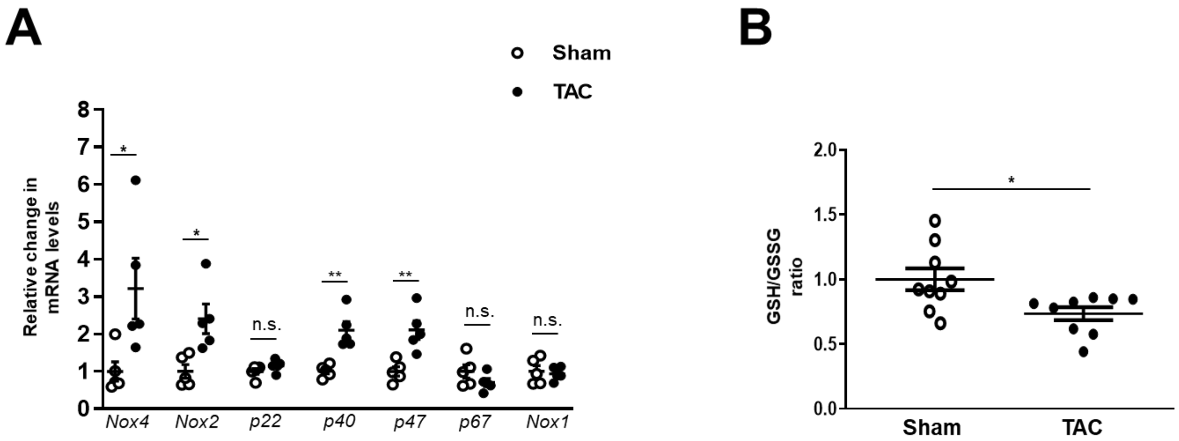

Suppl. Figure 2:

LV gene expression of NADPH oxidase subunits and glutathione redox state in WT hearts after chronic pressure overload. A: LV mRNA levels of Nox4, Nox2, Nox subunits ($p22^{phox}$, $p40^{phox}$, $p47^{phox}$ and $p67^{phox}$) and Nox1 normalized to GAPDH after TAC (transverse aortic constriction) compared to respective Sham controls. (n=5/group). **B:** Reduced (GSH) versus oxidized (GSSG) glutathione ratio in LV lysates after TAC compared to Sham controls. (n=9/group). * $p<0.05$, ** $p<0.01$, n.s.: not significant between Sham and TAC by unpaired Student´s t-test.

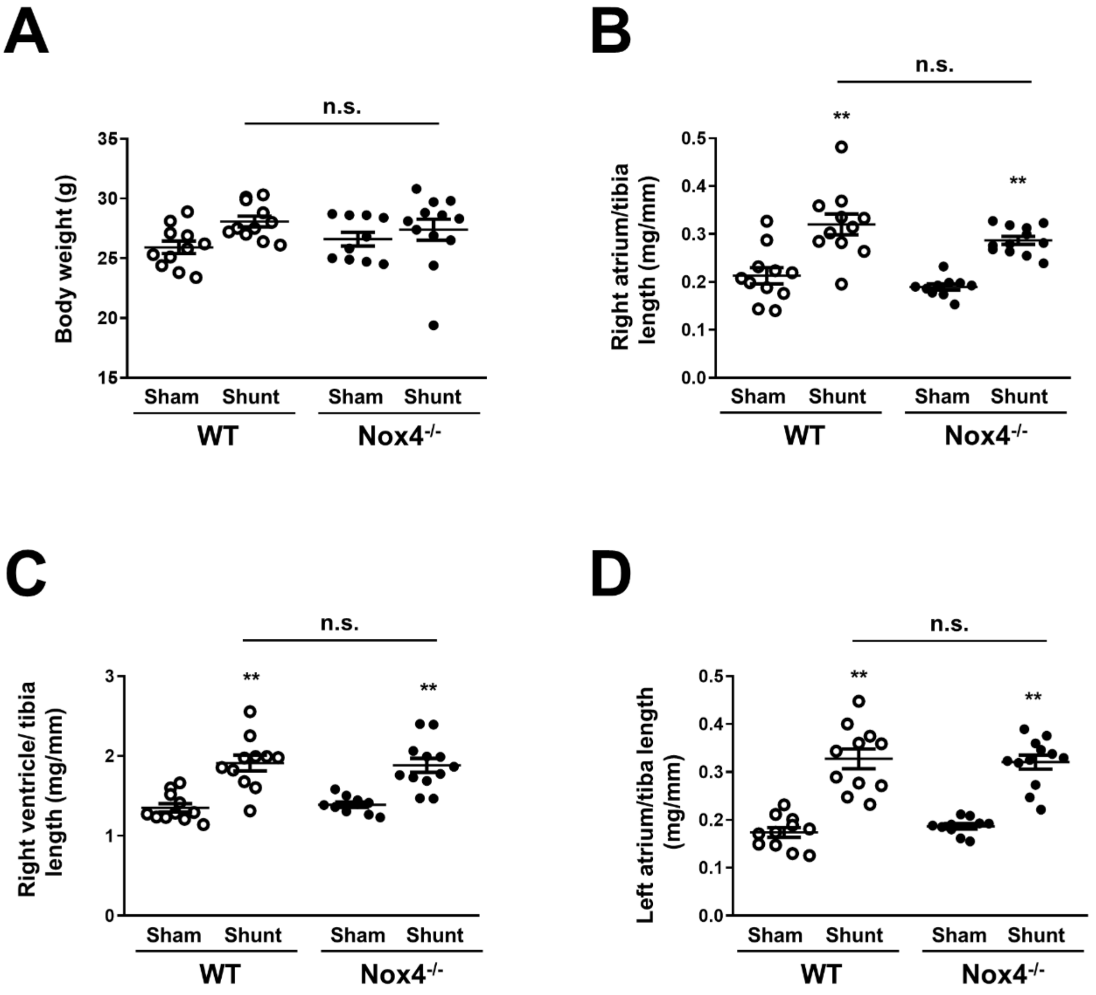

Suppl. Figure 3:

Morphological data in Nox4$^{-/-}$ mice and WT littermates after volume overload. A: Total body weight, **B:** right atrial weight (RAW), **C:** right ventricular weight (RVW), **D:** left atrial weight (LAW) versus tibia length (TL). (n=10-12/group). ** p<0.01 for Shunt versus respective Sham controls, n.s.: not significant between genotypes using two-way ANOVA followed by Bonferroni post-hoc test for multiple comparisons.

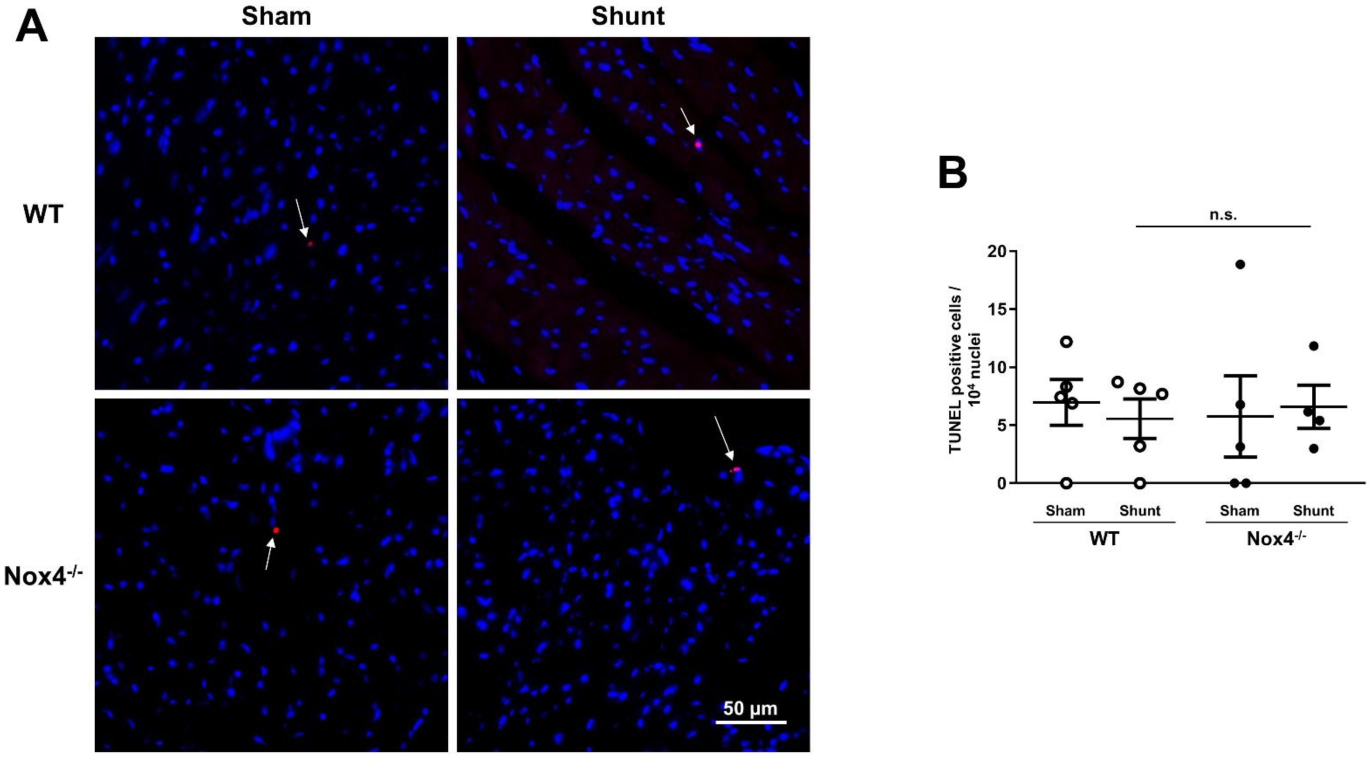

__Suppl. Figure 4:__

Left ventricular apoptosis in Nox4$^{-/-}$ mice and WT littermates following volume overload. Apoptotic cells in hearts were detected via TUNEL staining. White arrows indicate TUNEL-positive, apoptotic cells in representative histological images (**A**), mean data for number of apoptotic cells per counted nuclei are shown in **B**. (n=4-5/group). n.s.: not significant between genotypes using two-way ANOVA followed by Bonferroni post-hoc test for multiple comparisons.

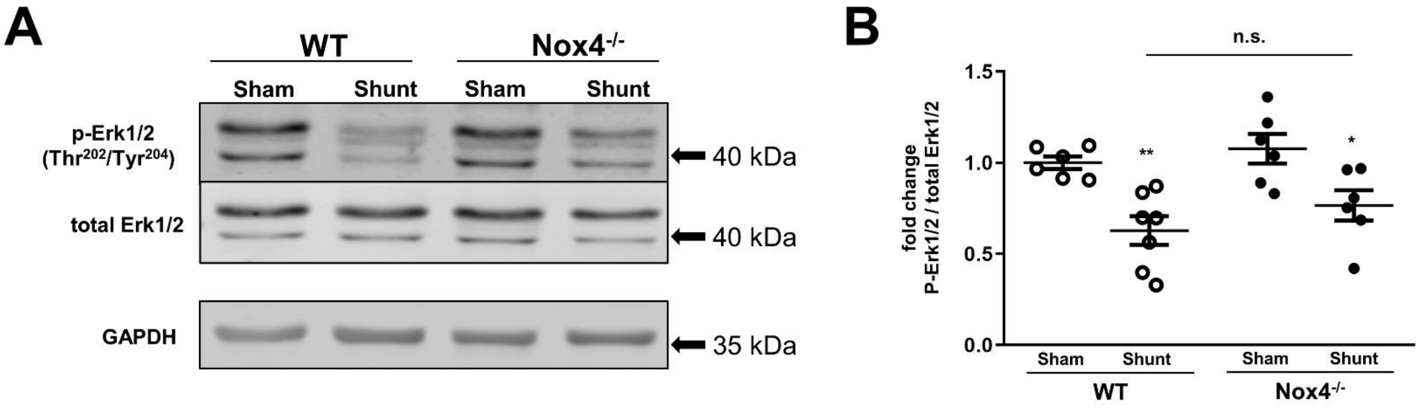

Suppl. Figure 5:

LV Erk1/2-phosphorylation in $Nox4^{-/-}$ mice and WT littermates following volume overload. A, Representative Western Blot images for phospho-Erk1/2 (p-Erk1/2) at Thr^{202}/Tyr^{204}, total Erk1/2 and GAPDH as loading control. **B,** mean data for p-Erk1/2 over total Erk1/2 ratio after Shunt and Sham. (n=6/group). * $p<0.05$, ** $p<0.01$ for Shunt versus respective Sham controls, n.s.: not significant between genotypes using two-way ANOVA followed by Bonferroni post-hoc test for multiple comparisons.

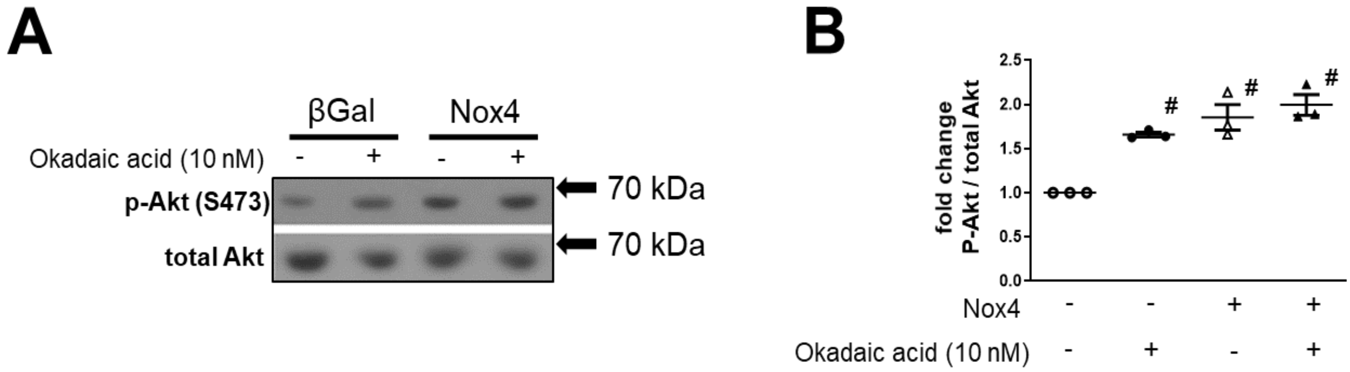

<u>Suppl. Figure 6:</u>

Effect of okadaic acid treatment on Nox4-dependent Akt phosphorylation in H9C2 cells. A,B: H9C2 cells overexpressing control βGal or Nox4 were treated with 10 nM okadaic acid or vehicle control for 30 minutes prior to lysis. Lysates were probed for phospho-Akt at Ser^{473} (p-Akt) and total Akt protein content by immunoblotting followed by densitometric quantification. (n=3 independent experiments). # p<0.01 vs respective control (-Nox4, - okadaic acid) using 1-way ANOVA followed by Bonferroni post-hoc test for multiple comparisons.

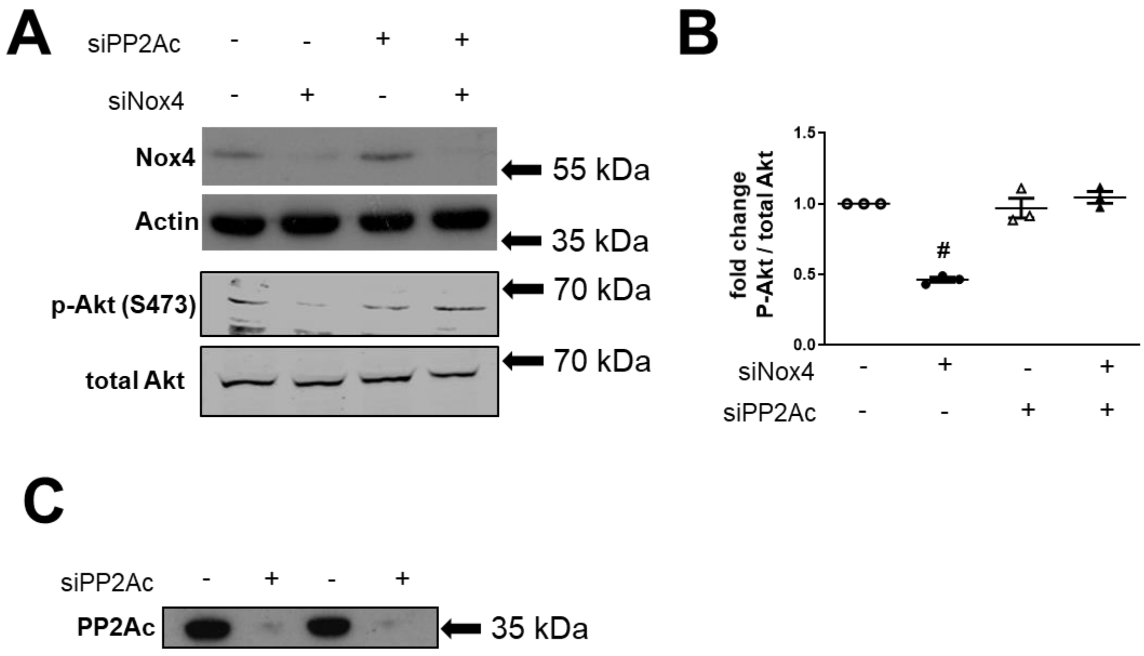

Suppl. Figure 7:

Nox4-dependent Akt phosphorylation involves PP2A in primary rat cardiomyocytes. A,B: Primary rat cardiomoyctes were treated with specific siRNAs targeting Nox4 (+siNox4) and/or the catalytic subunit of PP2A (+siPP2Ac), and respective controls (-siNox4, -siPP2Ac). Protein levels of Nox4, actin, phospho-Akt (Ser^{473}) and total Akt were assessed by immunoblotting followed by densitometric quantification. (n=3 independent experiments). # p<0.01 vs all three other conditions using 1-way ANOVA followed by Bonferroni post-hoc test for multiple comparisons. **C:** Verification of siRNA-mediated knockdown of PP2Ac in untreated H9C2 cells.

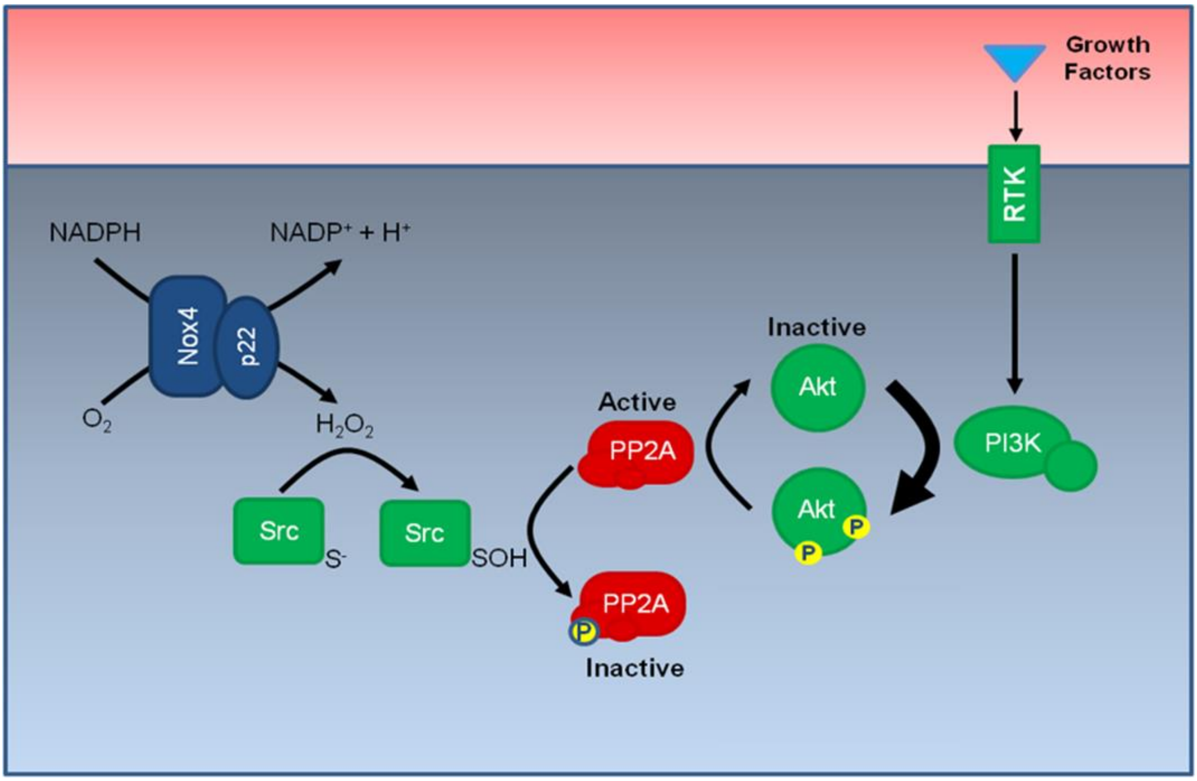

<u>**Suppl. Figure 8:**</u>

Nox4 maintains Akt phosphorylation by Src-mediated inactivation of protein phosphatase 2A (PP2A) in cardiac cells. Schematic illustrating the mechanism underlying enhancement of Akt activation by Nox4. Redox activation of Src kinase by Nox4 leads to phosphorylation and inactivation of PP2A, which in turn enhances Akt phosphorylation.

Suppl. Table 1:

Primer sequences used for qRT-PCR.

Primer Set	Forward (5´-3´)	Reverse (5´-3´)
Acta1 **(α-skeletal actin)**	ATGCTTCTAGGCGCACTCGCGT	CACGTCAAAAACAGGCGCCGG
Atp2a2 **(Serca-2α)**	GTCTCCACATTTCTCTGCAAAATG	TAGAGCAATCTGGCCACTTACAAC
Cyba **(p22phox)**	TGCCCTCCACTTCCTGTT	GCAGATAGATCACACTGGCAAT
Cybb **(Nox2)**	ACTCCTTGGGTCAGCACTGG	GTTCCTGTCCAGTTGTCTTCG
Gapdh	ATGACAACTTTGTCAAGCTCATTT	GGTCCACCACCCTGTTGCT
Ncf1 **(p47phox)**	GGACACCTTCATTCGCCATA	CTGCCACTTAACCAGGAACAT
Ncf2 **(p67phox)**	TTGAACCTGTCACACAGCAAT	CCAGCACACACACAAACCTT
Ncf4 **(p40phox)**	CTGCTTTTCTGACTACCCACAG	AAGCTGCTCAAAGTCGCTCT
Nox1	CGCTCCCAGCAGAAGGTCGTGATTACCAAGG	GGAGTGACCCCAATCCCTGCCCCAACCA
Nox4	CCGGACAGTCCTGGCTTATC	TGCTTTTATCCAACAATCTTCT
Nppa **(ANP)**	CGTGCCCCGACCCACGCCAGCATGGGCTCC	GGCTCCGAGGGCCAGCGAGCAGAGCCCTCA
Nppb **(BNP)**	AAGGGAGAATACGGCATCATTG	ACAGCACCTTCAGGAGATCCA
